# ASO Author Reflections: Risk-Adapted Use of Adjuvant Systemic Therapy in Real-World Kidney Cancer Care

**DOI:** 10.1245/s10434-026-20472-z

**Published:** 2026-08-28

**Authors:** Maximilian Filzmayer, Michele Petix, Leonardo Quarta, Filippo Orlandi, Jordan A. Goyal, Luca Carmignani, Alberto Briganti, Salvatore Micali, Shahrokh F. Shariat, Mike Wenzel, Yasmine Heß-Busch, Clara Humke, Fred Saad, Felix K.-H. Chun, Pierre I. Karakiewicz

**Affiliations:** 1 Cancer Prognostics and Health Outcomes Unit, Division of Urology, University of Montreal Health Center, Montreal, Canada; 2 Department of Urology, Goethe University Frankfurt, University Hospital, Frankfurt am Main, Germany; 3 Università degli Studi di Milano, Milan, Italy; 4 Department of Urology, IRCCS Policlinico San Donato, Milan, Italy; 5 Vita-Salute San Raffaele University, Milan, Italy; 6 Division of Experimental Oncology/Unit of Urology, Urological Research Institute, IRCCS San Raffaele Scientific Institute, Milan, Italy; 7 Department of Urology, AOU di Modena, University of Modena and Reggio Emilia, Modena, Italy; 8 Department of Urology, Comprehensive Cancer Center, Medical University of Vienna, Vienna, Austria; 9 Department of Urology, Weill Cornell Medical College, New York City, NY USA; 10 Department of Urology, University of Texas Southwestern Medical Center, Dallas, TX USA; 11 Hourani Center of Applied Scientific Research, Al-Ahliyya Amman University, Amman, Jordan

## Past

Patients with adverse pathological features remain at high risk of recurrence after nephrectomy for kidney cancer, prompting the development of adjuvant systemic therapies (AST).^1^ Following regulatory approval and guideline endorsement, AST has become a standard option for selected high-risk patients.^2^^,^^3^ However, real-world AST use in eligible patients remains insufficiently characterized. In particular, population-based utilization in the post-approval era and whether treatment allocation reflects established histopathological high-risk features remain unclear.^4^

## Present

Our population-based study examined 6497 patients with clear-cell renal cell carcinoma who were eligible for AST after nephrectomy between 2018 and 2022.^5^ Overall, only 18% received AST, although utilization increased substantially over time, from 13% in 2018 to 36% in 2022. Treatment allocation was risk-adapted: T4 stage, tumor grade 4, and lymph-node involvement independently predicted AST use. Moreover, AST utilization increased stepwise with the accumulation of these features, from 14% among patients without any of them to 40% among those harboring all three.

These findings suggest that adverse pathological characteristics appropriately influence treatment decisions in routine practice. Nevertheless, AST remained infrequently used, even among patients at the highest risk of recurrence. This observation may indicate incomplete implementation of emerging evidence but should not be equated with inappropriate care. Registry data cannot capture comorbidity, contraindications, patient preferences, or shared decision-making, and AST should be offered selectively rather than administered universally.

## Future

Future studies should evaluate treatment patterns and real-world oncological outcomes as more mature follow-up in the era of adjuvant immune checkpoint inhibition becomes available. Data sources that distinguish between specific systemic agents and capture recurrence, comorbidity, contraindications, and patient preferences will be required to better assess treatment allocation and effectiveness. In parallel, greater awareness of guideline recommendations among treating physicians and multidisciplinary decision-making may help optimize the risk-adapted use of AST in routine clinical practice.
